# Idiopathic young-onset Fahr’s disease with schizophrenia-like presentation: a case report

**DOI:** 10.3389/fpsyt.2024.1391607

**Published:** 2024-05-21

**Authors:** Wen-Cheng Li, Ya-Chu Hsieh, Pei-Ting Chen, Chia-Ning Lee, Tsung-Yu Tsai

**Affiliations:** Department of Psychiatry, National Cheng Kung University Hospital, College of Medicine, National Cheng Kung University, Tainan, Taiwan

**Keywords:** adolescent psychosis, basal ganglia calcification, aggression, schizophrenia, Fahr’s disease

## Abstract

This case report describes an exceptionally rare case in which a prior diagnosis of schizophrenia was later determined to be early-onset Fahr’s disease, linked to a genetic mutation in the SLC20A2 gene. Initially, the patient exhibited symptoms resembling schizophrenia, including aggression and hostility, and was highly susceptible to medication side effects such as restlessness and Parkinsonism. Despite maintaining independent activities of daily living, his neurological examinations revealed hidden weakness on the left side. Following adjustments to the medication regimen, stability was achieved with residual psychotic symptoms under treatment with Risperidone 1.5mg/day, Valproic acid 1500mg/day, and Quetiapine 37.5mg/day. This case underscores the importance of conducting comprehensive imaging studies at the time of initial psychiatric diagnosis, regardless of the apparent typicality of the presentation. Additionally, it emphasizes the need for patience and adherence to the “Start Low and Go Slow” approach in medication management to minimize the risk of exacerbating psychiatric symptoms and aggression.

## Introduction

Fahr’s disease is a rare neurodegenerative disorder with a prevalence of <1/1,000,000 and commonly affects people aged 40s and 50s. Fahr’s disease is characterized by idiopathic calcification of the bilateral basal ganglia and striopallidodentate area. The clinical presentation of this disease varies and may include psychosis, cognitive impairment, seizures, headaches, and motor function deterioration (1). The optimal treatment for Fahr’s disease remains unclear (2). While antipsychotics can offer symptomatic management of psychosis, patients with Fahr’s disease are more susceptible to side effects such as extrapyramidal syndromes (3). Achieving a balance between treatment and medication-related adverse effects is crucial in helping patients control symptoms and preserve their functioning. In this report, we present a case of early-onset Fahr’s disease that initially presented with symptoms resembling schizophrenia and further discuss the treatment experience and response. The case report has been approved by the Institutional Review Board, National Cheng Kung University Hospital (B-EC-113–002)

## Case

The 20-year-old unmarried male, diagnosed with schizophrenia at 17, was hospitalized due to psychotic manifestations, characterized by auditory hallucinations, persecutory delusions, disorganized thoughts, and violent behaviors with no history of mental, genetic, or neurological disorders. Additionally, the patient had neither family history of similar complaints nor documented history of Parkinsonism, infectious etiologies, or substance abuse at the time of initial diagnosis. The patient maintained modest academic performance during the period spanning from 17 to 19 years while experiencing mild auditory hallucination and paranoid ideation, managed with a regimen of Paliperidone at a dosage of 3mg per day. However, a relapse of psychotic symptoms occurred following the discontinuation of medication without medical consultation, prompting the subsequent adjustments in treatment modalities. Initially, Paliperidone at 3mg daily for one month yielded a suboptimal response, leading to the transition to a combination regimen comprising Aripiprazole at 10mg daily alongside Biperiden at 2mg daily, commenced two months preceding the current admission. Moreover, Fluoxetine at 10mg daily was introduced 1.5 months before the admission, because of compulsive hand washing. Further alterations in pharmacotherapy included the incorporation of Risperidone oral solution at 1mg daily for one month, in conjunction with Flupentixol Decanoate at 20mg administered once biweekly. Upon admission, the patient was receiving Aripiprazole at 10mg daily, Fluoxetine at 10mg daily, Risperidone at 2mg daily, Flupentixol Decanoate at 20mg biweekly, and Biperiden at 2mg daily. Despite the multifaceted pharmacological approach, symptomatic relief remained elusive, with concurrent escalation of aggressive behaviors.

**Figure 1:**
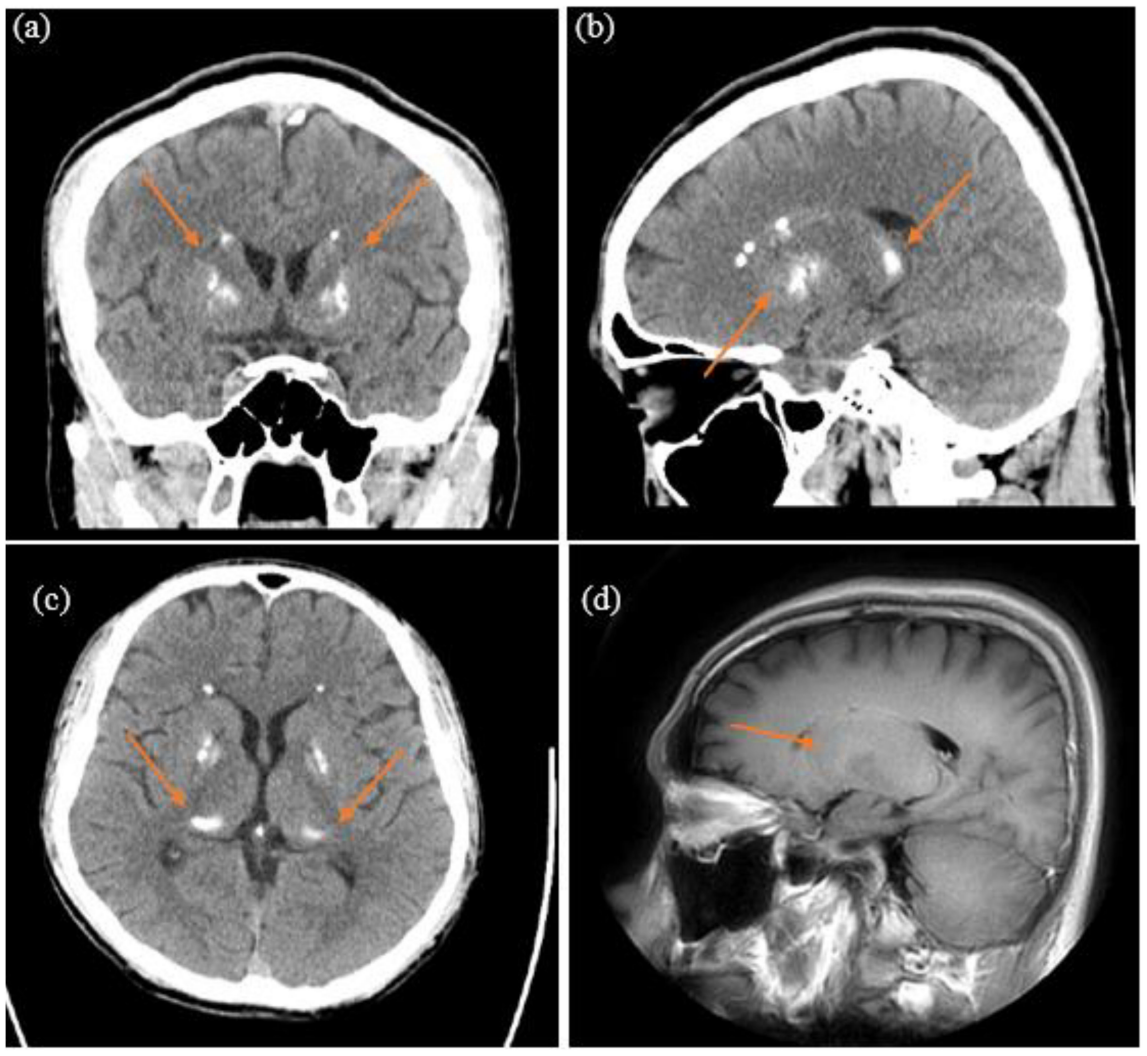
Basal ganglia calcification in the brain computed tomography: **(A)** coronal plane; **(B)** sagittal plane; **(C)** transverse plane. Basal ganglia calcification in the brain magnetic resonance imaging: **(D)** sagittal plane.

Throughout his hospitalization, the patient exhibited concealed left-side weakness and mild muscle rigidity. He maintained a stable gait and independent activities of daily living. Neurological examinations and physical examinations showed negative findings except for the aforementioned. Brain computed tomography (CT) and magnetic resonance imaging (MRI) showed calcifications in the bilateral globus pallidus, caudate nucleus, thalami, and subcortical areas, with no evident vessel abnormalities (**Figure 1**). Notably, the patient displayed marked aggression and hostility and exhibited pronounced susceptibility to medication-related adverse effects, such as restlessness and Parkinsonism. To optimize therapeutic efficacy while mitigating risks, the medication regimen was transitioned from Aripiprazole (5–10mg/day) to Olanzapine (7.5mg/day; administered for a total of 18 days), and ultimately to Risperidone (1mg/day). The patient’s blood investigations including ceruloplasmin, vitamin D, calcium, magnesium, phosphate, thyroid and parathyroid hormone, and alkaline phosphatase showed no abnormalities. Therefore, given the poor response to the patient’s psychotic and aggressive behaviors, and high susceptibility to psychotropic adverse effects, with supportive brain CT and MRI findings, a diagnosis of Fahr’s disease was considered. The revised diagnosis was further confirmed by genetic testing unveiling a deficit in the SLC20SA gene. Following four weeks of treatment adjustment, the patient was stabilized on a regimen comprising Risperidone 1.5mg/day, Valproic acid 1500mg/day, and Quetiapine 37.5mg/day. Upon discharge from the psychiatric acute ward, the patient had mild psychotic symptoms devoid of disruptive behaviors or violence. His Cognitive Ability Screening Inventory (CASI) score, which might be underestimated because of psychotic symptoms, was 75/100. The component scores of CASI were as follows: Short-term memory: 9/12, Long-term memory: 7/10, Attention: 3/8, Mental manipulation and concentration: 8/10, Orientation: 18/18, Abstract thinking and judgment: 6/12, Language: 8/10, Visual construction and drawing: 10/10, and Fluency: 6/10.

From the patient’s perspective, as assessed through the Health, Personality, and Habit Test -A Form Scale, he continued to harbor beliefs that he was being followed, spied on, and subjected to potential harm or attack. He perceived a sense of loss of autonomy, with the conviction that others had control over his body and were privy to his thoughts. Despite recognizing the severity and progressive nature of his mental disorder, he expressed feelings of frustration and sadness regarding his experiences. Acknowledging the necessity for long-term treatment, the patient consented to transfer to a psychiatric chronic ward to facilitate disease management and rehabilitation.

## Discussion

Fahr’s disease is a neurodegenerative disorder that can occur as a secondary manifestation of hereditary or endocrine diseases. The most common site of abnormal calcium deposits is the globus pallidus. Additionally, deposits have been observed in the globus pallidus dentate nuclei, thalami, and other deep cortical structures (2, 4–6). Fahr’s disease can manifest at any age, but the most frequent onset age is between the 30s and 50s (2, 6, 7). The main clinical presentations include neurological symptoms such as seizures, Parkinsonism, rigidity, ataxia, dysarthria, tremor, and headaches, as well as psychiatric symptoms such as mood disorders, psychotic symptoms, irritability, aggressiveness, lack of impulse control, regressive behaviors, behavioral disinhibition, obsessive-compulsive spectrum symptoms, and cognitive and psychomotor impairment. Extrapyramidal symptoms are observed in 50–56% of initial presentations, while psychiatric symptoms such as psychosis, mania, anxiety, and apathy contribute to 40% (3, 7–9). Two clinical variants of Fahr’s disease were proposed by other studies: early onset and late onset (10). In the early onset variant, psychiatric symptoms were found to present earlier to movement abnormalities. While the late-onset variant tends to be an initial presentation with severe movement disorder followed by dementia-like symptoms. The present case echoed this finding from the literature. He had no neurological signs and movement abnormalities in the background of florid psychosis. Moreover, instead of muscle rigidity, he was more susceptible to akathisia mimicked as worsening psychotic symptoms and aggression.

Currently, there is no consensus on treating Fahr’s disease and its associated symptoms. The prognosis of Fahr’s disease is variable and unpredictable (2). Most of the literature primarily focuses on managing and controlling the symptoms. According to early-onset case reports, low doses of Risperidone and Lithium (11, 12) might be effective for psychotic symptom control. Based on the present case’s experience, a low dose of Risperidone might be a better option in treating Fahr’s disease. Other case reports on adults and older adults, various medications have been suggested, including antipsychotics such as Haloperidol 10mg/day (4, 13), Risperidone 1–4mg/day (4, 6, 7, 14, 15), Olanzapine 5mg/day (6, 14), Amisulpride 100–200mg/day (7, 16), Aripiprazole 10mg/day (8, 17), Quetiapine 25–75mg/day (8, 18), and Clozapine (13, 19). Mood stabilizers such as Lithium 900mg/day (4, 12), Valproic acid 600–1000mg/day (7, 14, 20), and Oxcarbazepine 300–600mg/day (8, 15) have also been suggested. Dopamine and a dopamine receptor agonist may relieve the extra-pyramidal symptoms of Fahr’s Disease (21).

The physical and occupational therapy programs, such as passive and active physiotherapy, muscle strengthening exercises, and independence training, integrated according to patients’ specific motor deficits may improve their mobility, preserve their functional independence, and enhance their quality of life (22).

In summary, Fahr’s disease is a neurodegenerative disorder presenting with various neuropsychiatric symptoms that are often underestimated. This case highlights two important concepts regarding the early onset of psychotic symptoms: (1) comprehensive differential diagnosis, emphasizing the need for thorough clinical assessment and meticulous consideration of various potential diagnoses, particularly when the patient has atypical presentation and unexpected response to therapy; and (2) individual variation in sensitivity to medication, suggesting the importance of initiating treatment with a low dose and gradually titrating it to achieve the optimal dosage through careful titration.

## Data availability statement

The original contributions presented in the study are included in the article/supplementary material. Further inquiries can be directed to the corresponding author.

## Ethics statement

Written informed consent was obtained from the individual(s) for the publication of any potentially identifiable images or data included in this article.

## Author contributions

W-CL: Writing – original draft, Writing – review & editing. Y-CH: Conceptualization, Validation, Writing – original draft. P-TC: Conceptualization, Writing – review & editing. C-NL: Conceptualization, Supervision, Validation, Writing – review & editing. T-YT: Conceptualization, Validation, Writing – original draft, Writing – review & editing.

## References

[1] Saleem S Aghemo K Salmanzadeh R DeAngelo O Salmanzadeh A. M . Fahr's syndrome: literature review of current evidence. Orphanet J Rare Dis. (2013) 8:156. doi: 10.1186/1750-1172-8-156 24098952 PMC3853434

[2] Amisha F Munakomi S . Fahr syndrome (2022). Available online at: https://www.ncbi.nlm.nih.gov/books/NBK560857/. 32809692

[3] Ghormode D Maheshwari U Kate N Grover S . Fahr's disease and psychiatric syndromes: A case series. Ind Psychiatry J. (2011) 20:136–8. doi: 10.4103/0972-6748.102527 PMC3530285 23271871

[4] Dennis AC Nwabueze C Banu F Nisenoff CD Olupona T . Bilateral basal ganglia calcifications manifesting as psychosis with manic features: A case report on fahr's syndrome. Cureus. (2023) 15:e34547. doi: 10.7759/cureus.34547 36879722 PMC9985407

[5] Bu W Hou L Zhu M Zhang R Zhang X Zhang X . SLC20A2-related primary familial brain calcification with purely acute psychiatric symptoms: a case report. BMC Neurol. (2022) 22:265. doi: 10.1186/s12883-022-02798-9 35850697 PMC9290231

[6] Mohapatra S Satapathy A . A case of schizophrenia like psychosis due to fahr's disease. Indian J Psychol Med. (2016) 38:155–6. doi: 10.4103/0253-7176.178813 PMC4820558 27114631

[7] Kumar P Singh R Shah K . Psychiatric manifestations in fahr's syndrome: A case report. Cureus. (2020) 12:e10770. doi: 10.7759/cureus.10770 33154842 PMC7606226

[8] Carbone MG Della Rocca F . Neuropsychiatric manifestations of fahr's disease, diagnostic and therapeutic challenge: A case report and a literature review. Clin Neuropsychiatry. (2022) 19:121–31. doi: 10.36131/cnfioritieditore20220206 PMC9112992 35601245

[9] König P . Psychopathological alterations in cases of symmetrical basal ganglia sclerosis. Biol Psychiatry. (1989) 25:459–68. doi: 10.1016/0006-3223(89)90199-6 2930811

[10] Aghemo K Salmanzadeh R DeAngelo O Salmanzadeh AM . Advanced early-onset fahr's disease: A case report. Cureus. (2023) 15:e39495. doi: 10.7759/cureus.39495 37362501 PMC10290546

[11] Naqvi S Arshad S Hanif R Elfert KAH . Fahr's syndrome misdiagnosed as schizophrenia: A case report. Cureus. (2017) 9:e1071. doi: 10.7759/cureus.1071 28473946 PMC5413360

[12] Munir KM . The treatment of psychotic symptoms in Fahr's disease with lithium carbonate. J Clin Psychopharmacol. (1986) 6:36–8. doi: 10.1097/00004714-198602000-00008 3081601

[13] Kane I Light M Khan M Osewa I Nobler M Siddiqi N . Acute psychosis with manic features in patient with Fahrs syndrome: A Case report and Clinical review. Neuropsychiatry. (2017) 07:254–7. doi: 10.4172/Neuropsychiatry

[14] Chhetri B Gyeltshen D Letho Z . Bipolar affective disorder in a patient with Fahr's disease: The first recorded case in Bhutan. SAGE Open Med Case Rep. (2022) 10:1–5. doi: 10.1177/2050313X221125324 PMC9486261 36147593

[15] Faye AD Gawande S Tadke R Kirpekar VC Bhave SH . A case of psychosis due to Fahr's syndrome and response to behavioral disturbances with risperidone and oxcarbazepine. Indian J Psychiatry. (2014) 56:188–90. doi: 10.4103/0019-5545.130506 PMC4040070 24891710

[16] Srivastava S Bhatia M Sharma V Mahajan S Rajender G . Fahr's disease: an incidental finding in a case presenting with psychosis. German J Psychiatry. (2010) 13:86–9.

[17] Evangelista J Rosales R . Acute ischemic stroke in a filipino with parkinsonian fahr’s disease: A case report. J Medicine Univ Santo Tomas. (2018) 2:220–3. doi: 10.35460/2546-1621

[18] Kono S Manabe Y Tanaka T Fujii D Sakai Y Narai H . A case of fahr's disease presenting as chorea successfully treated by the use of quetiapine. Clin Med Case Rep. (2009) 2:63–5. doi: 10.4137/CCRep.S3423 PMC3785361 24179377

[19] Fekih-Romdhane F Tounsi A Fadhel SB Ridha R . Fahr's syndrome revealed by psychiatric disorders. L'information psychiatrique. (2020) 96:279–83. doi: 10.1684/ipe.2020.2097

[20] Ghogare AS Nemade S . Fahr's syndrome presenting as pre-senile dementia with behavioral abnormalities: A rare case report. Cureus. (2021) 13:e20680. doi: 10.7759/cureus.20680 35106221 PMC8786577

[21] Wang H Shao B Wang L Ye Q . Fahr's disease in two siblings in a family: A case report. Exp Ther Med. (2015) 9:1931–3. doi: 10.3892/etm.2015.2356 PMC4471777 26136916

[22] Tuncer D Çınar B Hanagasi H . Physiotherapy and rehabilitation in Fahr’s disease: A case report. IGUSABDER (2023) 19:334–9. doi: 10.38079/igusabder.1183826

